# Adsorption Characteristics of Indigenous Chromium-Resistant *Aspergillus niger* Strain Isolated from Red Soil for Remediation of Toxic Chromium in Red Soil Environments

**DOI:** 10.3390/toxics11010031

**Published:** 2022-12-29

**Authors:** Jiwei Xu, Lumeng Li, Huabin Wang, Zhanyuan Gao, Chuanshu Wang, Rong Sun, Yong Zhang, Wumei Xu, Xiying Hou, Rui Xu

**Affiliations:** 1 School of Energy and Environment Science, Yunnan Normal University, Kunming 650500, China; 2 Provincial Key Laboratory of Rural Energy Engineering, Kunming 650500, China

**Keywords:** heavy metal, mechanism, adsorption kinetics, microbial remediation, state transformation

## Abstract

The microbial treatment of soil has great potential to reduce chromium pollution. Here, an indigenous chromium-resistant *Aspergillus niger* strain (A1) was isolated and screened from heavily chromium-contaminated red soil in Yunnan Province, China using a traditional isolation method and a selective culture experiment. The molecular identification of A1 was achieved using 18S rRNA sequencing. The tolerance of the strain to toxic chromium was evaluated through pure laboratory culture. The adsorption effect and mechanism of A1 on chromium in red soil were further studied. The study concluded that A1 exhibited strong activity with exposure to 500 mg·L^−1^ Cr^6+^. Chromium adsorption by *A. niger* occurred mainly through intracellular metabolism, surface complexations with EPS, and chemical reduction with -C=C-, -OXuH, NH_2_, and -C=0. The optimized results showed that A1 had the best Cr^6+^ removal effect at pH 4, 40 °C, and a 60 h culture time. Compared with the inoculating of exogenous microbial agents, after inoculating A1 into the chromium-contaminated red soil, Cr^6+^ content was significantly reduced, and the high-toxicity chromium state (water-soluble and exchange states) decreased, whereas the low-toxicity chromium state (precipitation and residue states) increased. The results of red soil ITS also showed that the inoculation of indigenous microorganisms can better colonize the red soil. This study proves the feasibility of the application of indigenous *A. niger* to address red soil chromium pollution and provides a new idea and theoretical support for red soil remediation.

## 1. Introduction

With rapid industrial development and agricultural modernization, heavy metals in soil have become serious agricultural pollutants and ecological risk factors [1,2]. Even trace concentrations of heavy metal can be toxic to organisms [3], and after being absorbed by plants, heavy metals can be accumulated up the food chain, increasing foods’ toxicity risks [4]. A soil environment is more complex than a solution environment, and it is difficult to determine toxicity directly via the valence of heavy metal elements. At the same time, heavy metal pollutants are difficult to extract from soil using conventional methods and cannot be degraded, except through transformation between different forms [5]. Thus, the deactivation of heavy metals into low-toxicity forms with low bioavailability is an important method to reduce the harm of heavy metals in soil.

Red soil is a common soil type that is mainly distributed in Africa, Asia, Oceania, South America, and tropical and subtropical areas of North America. Red soil formation occurs primarily due to the interaction of vigorous bioaccumulation, bioclimate factors, desiliconization, iron rich aluminization, and weathering processes [6]. Therefore, red soil is usually acidic to strongly acidic and rich in exchangeable acids [7]. Heavy metals (such as chromium and nickel) are prone to forming water-soluble and exchangeable states with extremely high mobility and toxicity in acidic environments, thereby increasing their ecological risks [8]. Yunnan Province in China is a concentrated area of red soil. The cultivated area of red soil is 49.36 million mu, accounting for about 60% of the cultivated area of the province and 73% of the land area. Yunnan is characterized by acidic red soil that is rich in iron and aluminum [9] and suitable for growing various crops; however, the high background value of heavy metals and the forms of heavy metals easily absorbed by plants under acidic conditions constitute potential safety hazards. Common heavy metals include chromium, cadmium, arsenic, and lead [10]. A survey showed that the enrichment concentrations of As and Cr in Yunnan red soil were higher than the background value and average concentration of national standard soil [11]. Additionally, red soil pollution has concealment and hysteresis. Air pollution and water pollution are generally relatively straight and can be perceived through the senses, and red soil pollution is often determined via soil sample analysis, crop testing, and even research on the impact of human and animal health. Harm caused by the generation of pollution from red soil usually takes longer, meaning red soil pollution is cumulative [12]. Compared with ordinary soil, it is more difficult for pollutants to migrate, diffuse, and dilute in soil. Chromium is one of the most toxic heavy metals, with serious carcinogenic and teratogenic properties [13]. Chromium in soil has different oxidation states: −2, −1, 0, +1, +2, +3, +4, +5, and +6 [14]. The valence states +3 and +6 are the main chromium forms in soil [15]. Cr^3+^ has high stability and low toxicity, is difficult to absorb by living organisms, is easily precipitated in soil environments [16], and mainly exists in precipitated and residual states. Cr^6+^, a common oxidized form of chromium oxyanions, is highly toxic and highly soluble in water [17]. Cr^6+^ has a strong migration ability in soil and is the main form of the water-soluble and exchange states of chromium. Chromium in cultivated soil can be absorbed by crops, entering the human food chain, and pollutes water bodies through surface runoff and leaching, further propagating the contamination. Therefore, when treating soil chromium pollution, attention should be paid to adsorption methods and drivers that can transform chromium to a low-toxicity valence state, which reduces the toxicity and migration ability of chromium in soil.

At present, chromium pollution in soil is mainly treated using conventional physical or chemical methods, super-enriched plants, or microbial methods [18]. However, these traditional methods can be costly, impractical, and ineffective [19]; some even produce toxic sludge, adding more harmful chemicals to the environment [20]. Super-enriched plants can effectively extract heavy metal elements from soil; however, the long recovery time and accompanying land resource occupation problems limit their practical application [21]. Microbial remediation has emerged in recent years as a method for passivating or adsorbing heavy metals using natural and low-cost biomass resources, such as fungi, bacteria, algae, and moss. Compared with other methods, microbial remediation has low operating costs, high removal efficiency, and no secondary pollution [22]. Microbial remediation technology is the use of soil or water in the natural flora or the genetic engineering of bacteria, in the right environment, using microbial adsorption, precipitation, or redox, or through microbial respiration or metabolic pathways using toxic compounds as growth energy, so as to achieve or reduce the concentration of pollutant repair technology [23]. According to the current literature, there are two main mechanisms of microbial remediation of soil. First, EPS containing carboxyl (R-COO-), phosphate (R-HPO_4_^-^), sulfhydryl (R-SH), amine (R-NH_3_^+^), phenol (R-C_6_H_4_OH), and hydroxyl (R-OH) functional groups improves its bioavailability and uses the bonding of EPS surface functional groups with heavy metals to improve its biosorption capacity [24]. Another mechanism is microbial oxidation and reduction. The environmental toxicity of different valence states of heavy metals varies greatly [25]. Microorganisms convert toxic heavy metals into non-toxic and relatively unavailable forms through redox reactions. This is of great significance in the remediation process of heavy metal-contaminated soil, especially in environments that other remediation technologies cannot reach. [26]. Considering the physical and chemical properties of acidic red soil and its worldwide distribution, screening indigenous chromium-resistant strains from red soil could help treat heavy metal pollution in red soil environments. Additionally, exogenous microorganisms are often difficult to colonize in a soil environment, resulting in unstable remediation effects. For example, Jorquera found that the exogenous microorganisms Bacillus-like phosphobacteria (BLP) were difficult to keep alive and colonize in soil without bio-fertilizer [27]. Ghiglione found that the wild strain colonizes maize rhizosphere soil more easily than the exogenous strain [28]. Therefore, this study screened indigenous chromium-resistant strains from red soil and proved the feasibility of activating indigenous resistant microorganisms to reduce chromium toxicity in red soil. At the same time, the mechanism of chromium passivation was studied to lay a foundation for subsequent method improvement.

In this study, indigenous chromium-resistant *Aspergillus niger* strains were isolated and purified from red soil with severe chromium pollution, and strains with strong chromium adsorption capacity were screened. The effects of different operating variables on chromium adsorption capacity were studied by optimizing the conditions. The mechanism by which *A. niger* adsorbs chromium from red soil was studied by analyzing the changes in its surface functional groups before and after chromium adsorption and the energy spectrum of *A. niger*-adsorbed chromium. The ability of indigenous *Aspergillus niger* to reduce chromium availability in red soil was also evaluated by studying the morphological changes of chromium in red soil after inoculation with *Aspergillus niger*. Finally, we used metagenomic technology to study the effects of inoculation with indigenous *Aspergillus niger* on red soil microflora and evaluated the practicability and ecological risks of this method. Our findings support the feasibility of using indigenous strains to reduce chromium pollution in red soil, thereby improving the application of microbial remediation.

## 2. Materials and Methods

### 2.1. Culture Medium and Reagents

SDAY medium [29] was used for the isolation and preservation of microorganisms from red soil. The reagents diphenylcarbazide, potassium dichromate (K_₂_Cr_₂_O_₇_), glucose, agar, peptone, and ethanol were analytically pure. Fresh ddH_2_O was used for the experimental work.

### 2.2. Preparation of Chromium-Contaminated Red Soil

The red soil used in this experiment was collected from farmland in the Chenggong District, Kunming City, Yunnan Province, China. After natural air drying, the soil sample was screened twice through 20-mesh screens. The organic matter content in the red soil was 5.4%, pH was 4.5, and no chromium was detected. The soil used for strain screening was weighed, 1 g·kg^−1^ Cr^6+^ was added, and the soil was then allowed to rest for 60 d. The chromium content of the red soil was 922.34 mg·kg^−1^ when the strains were tested and screened. Sterile soil was prepared through sterilization at high temperature, followed by the addition of 400 mg·kg^−1^ of Cr^6+^.

### 2.3. Isolation of Chromium-Resistant Strains

Ten grams of soil was weighed for strain screening, and placed into a 250 mL conical flask, and 90 mL of ddH_2_O was added to it. The mixture was then shaken at 100 r·min^−1^ for 15–30 min and mixed thoroughly to obtain a soil suspension. Under sterile conditions, 1 mL of the soil suspension was removed and 9 mL of ddH_2_O was added. This mixture was mixed well; 1 mL of the mixture was removed and 9 mL of ddH_2_O was added, and this was repeated five times to obtain 10^−2^, 10^−3^, 10^−4^, and 10^−5^ dilutions. Then, 0.1 mL of each dilution of each concentration was taken, spread on SDAY plates, and incubated at 37 °C for 5 d. During the culturing period, colonies with evident differences were selected, scribed, and purified on the improved SDAY plate. After several repetitions, the strain was preserved in test tube slant medium at 4 °C.

### 2.4. Selection of Strains with Chromium Adsorption Capacity

The fungi obtained via screening were numbered A1–A7, and the bacteria were numbered B1–B3. The screened chromium-resistant strains were cultured in a liquid medium containing 50 mg·L^−1^ or 500 mg·L^−1^ Cr^6+^ for 10 d. The Cr^6+^ concentration in the culture medium was measured at the end of the culturing period, and the Cr^6+^ adsorption rates of different strains were calculated. The strain with the strongest adsorption capacity was screened. 

### 2.5. Identification and Phylogenetic Analysis of Strains

The screened fungi were identified via 18S rRNA sequencing. DNA was extracted according to standard procedures and analyzed via agarose gel electrophoresis. Fungal ITS1 and ITS4 fragments were amplified through polymerase chain reaction (PCR). A 25 μL PCR reaction system containing 5 μL strain DNA (50 mg·L^−1^) with ITS1 and ITS4 primers, 12.5 μL Taq Master Mix, and 7.5 μL RNase-Free Water was used and mixed well, and then, 5 μL mineral oil seal was applied. After denaturation (94 °C, 4 min), PCR was performed for 35 cycles under the following thermal conditions: (1) denaturation (94 °C, 45 s), (2) annealing (55 °C, 35 s), and (3) extension (72 °C, 1 min). The amplified products were sequenced after the specified PCR cycle. Fungal sequences were compared with other species sequences using the NCBI blast tool. Phylogenetic analysis between the amplified product and its relatives was carried out using MEGA11.

### 2.6. A. niger Colony Characteristics under Chromium Stress

The screened *A. niger* was inoculated on the modified SDAY plate containing Cr^6+^ concentrations of 0, 50, 100, 200, and 500 mg·L^−1^ using single-spore isolation, with three biological replicates. The diameters of single colonies under different Cr^6+^ concentrations were measured at 24 h intervals. *A. niger* was placed into a liquid medium at 30 °C and 150 r·min^−1^ for 24 h. Then, the liquid medium was inoculated into the liquid medium with Cr^6+^ concentrations of 0 (control (CK)), 100, and 500 mg·L^−1^ at a rate of 2%. The spore yield was measured every 3 h. After the experiment, *A. niger* bodies (in 500 mg·L^−1^ Cr^6+^) were filtered and collected. Fourier transform infrared spectroscopy (FTIR) was used to analyze changes in the surface functional groups of *A. niger* before and after contact with Cr^6+^. The energy spectrum of *A. niger* adsorbed chromium was analyzed via X-ray photoelectron spectroscopy (XPS).

### 2.7. Optimization Studies

After inoculating *A. niger* in liquid medium at 30 °C and 150 r·min^−1^ for 24 h, washing with sterile water, and filtering twice with four layers of gauze, a 6.7·10^6^/mL spore suspension was prepared for later use. For the optimization study, the following experiments both added 5 mL spore suspension (6.7·10^6^/mL) and were carried out in Erlenmeyer flasks under different conditions: pH (4–9), culture temperature (10–50 °C), contact time (18–72 h), and initial Cr^6+^ concentration (10–150 mg·L^−1^). The concentration of unabsorbed Cr was calculated as follows:

Cr^6+^
*removal rate* = (*c*_0_ − *c*_*e*_)/*c*_0_ × 100%, (1)

Cr^6+^
*ratio in solution* = *c*_*e*/_*c*_*t*_ × 100% (2)

where *c_o_*, *c_e_*, and *c_t_* are the initial and final Cr^6+^ concentrations (mg·L^−1^) and total Cr concentrations (mg·L^−1^) in solution, respectively.

The unabsorbed chromium was examined using atomic absorption spectroscopy. The concentration of Cr^6+^ was examined using the diphenylcarbazide spectrophotometric method.

### 2.8. Kinetic and Isotherm Validation

The relationship between the Cr^6+^ distributions in solution and *A. niger* was studied via adsorption kinetic analysis. Adsorption characteristic types were verified using pseudo-first-order models and pseudo-second-order models. The adsorption process was verified using models including Langmuir models, Freundlich models, and intra-particle diffusion models. Using the model-fitting tool in Origin 2021, the isotherm parameters and correlation coefficient R^2^ were calculated. Kinetic analysis was used to explain the mechanism of Cr^6+^ removal by *A. niger*. Compared with the Langmuir isotherm, the Freundlich isotherm described multilayer and heterogeneous adsorption. Therefore, the adsorption type of chromium ions by *A. niger* could be determined by comparing the fitting degree of the following five equations: 

Pseudo-first-order:

(3) $$\ln(q_{e}-q)=\ln q_{e}-K_{1}t,$$

where *qe* (mg/g) is the equilibrium adsorption capacity; *qt* (mg/g) is the adsorption capacity at t time; and *K*_1_ is the pseudo-first-order constant (h^−1^).

Pseudo-second-order:

(4) $$\;\;\;\;\frac{1}{q_{e}-q}=\frac{1}{q_{e}}+K_{2}t,$$

where *K*_2_ is the pseudo-second-order constant (g/mg/h).

Langmuir model:

(5) $$\;\;q_{e}=q_{max}\times \frac{K_{L}C_{e}}{1+K_{L}C_{e}},$$

where *q_max_* is the Langmuir monolayer capacity (mg/g) and *K_L_* is the Langmuir constant (L/mg).

Freundlich model:

(6) $$\;q_{e}=K_{F}C_{e}^{1/n},$$

where *n* is the Freundlich exponent and *K_F_* is the Freundlich constant ((mg/g)/(L/mg)^(1/n)^).

Intra-particle diffusion model (Weber-Morris equation) (Martins et al., 2015):

(7) $$q_{t}=K_{i}t^{1/2}+C_{i},$$

where *K_i_* (mg·g^−1^·h^−1/2^) is the intra-particle diffusion rate of the adsorption process at any stage and *C_i_* is the intercept describing the effects of the boundary layer thickness.

### 2.9. Effects of A. niger Inoculation Amount on Chromium State Transformation

A total of 200 g of the chromium-containing soil was weighed into a 250 mL beaker and inoculated with 3 mL and 10 mL (M3 and M10, respectively) of *A. niger* culture solution (6.7·10^6^/mL spore suspension) that had been cultivated for 48 h; each treatment was repeated three times. During the experiment, sterile water was added to maintain soil moisture at ~60%. Taking sterile soil as a blank control (CK) and sterile soil inoculated with 3 mL commercial microbial agent (6.7·10^6^/mL spore suspension) as a control (B-CK), 10 g soil samples were taken at 0, 5, 10, 20, and 30 d after inoculation. 

After freeze-drying, crushing, and screening, 5 g of the collected soil samples were placed in a 250 mL beaker; then, 50 mL alkaline extraction solution (Na_2_CO_3_-NaOH mixed solution, pH > 11.5) and 400 mg MgCl_2_ and 0.5 mL K_2_HPO_4_-KH_2_PO_4_ buffer solution were added. After sealing with polyethylene film, it was stirred and heated to 95 °C for 60 min. After cooling and filtration, pH was adjusted to 7.5 ± 0.5 with HNO_3_, and the concentration of Cr^6+^ was determined via atomic absorption spectrophotometry.

The content of different forms of Cr in soil was determined using the sequential extraction method. Firstly, the 5 g red soil was fully soaked, and the concentration of water-soluble chromium in the leached solution was detected after extraction and filtration. Then, for the exchange, precipitation (Fe-Mn combined), and organic-bound states of chromium, 1 mol·L^−1^ NH_4_Ac, 2 mol·L^−1^ HCl, and 5% H_2_O_2_-2 mol·L^−1^ HCl were used as the extraction agents, respectively. Finally, the residual state was determined after the 0.2 g residual solid was fully digested by the mixed acid (1.19 g/mL HCl: 1.42 g/mL HNO_3_: 1.49 g/mL HF = 3:6:2) in a Microwave Digestion System. The above steps were completed in an artificial climate chamber at 25 ± 0.1 °C. The ratio of each extraction agent to soil was 5:1. The single extraction time was 4 h (oscillating for 2 h, standing for 2 h). The unabsorbed chromium was examined using atomic absorption spectroscopy; the concentration of Cr^6+^ was examined using the diphenylcarbazide spectrophotometric method.

### 2.10. Analysis of Red Soil Fungus Diversity after A. niger Inoculation

Total DNA was extracted from the red soil treated with CK and M10 on the 30th day. PCR amplification was performed, and the products were purified, quantified, and homogenized to form a sequencing library. The library was sequenced using Illumina Novaseq 6000 after passing quality inspection, and the original image data files obtained were transformed into Sequenced Reads via Base Calling analysis. Sequenced Reads were filtered by low-quality filtering length; then, OTUs/ASVs were obtained via clustering and denoising. Species classification was performed according to the sequence composition of the feature, and a Wayne diagram was drawn.

## 3. Results

### 3.1. Isolation and Identification of Chromium-Resistant Strains

**Figure 1:**
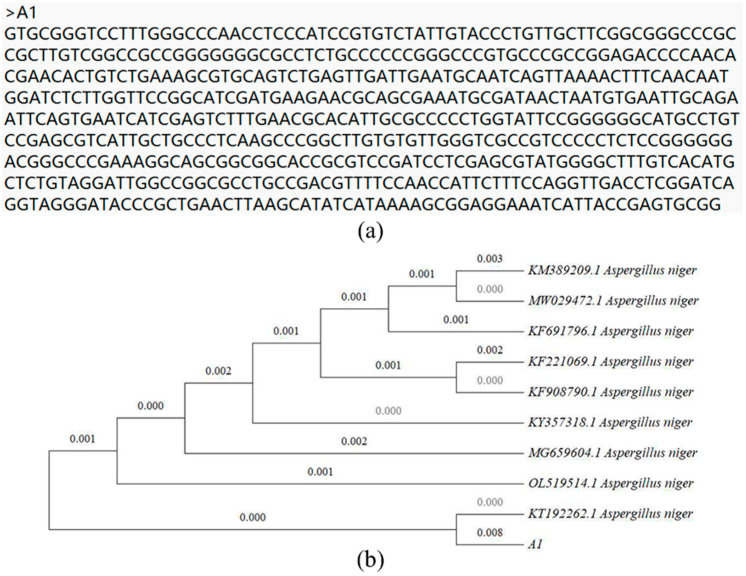
(**a**) Sequencing result of A1. (**b**) Phylogenetic tree of A1.

Ten strains with high activity were screened from chromium-contaminated soil. The results obtained after screening with liquid medium containing Cr^6+^ are provided in Table 1. A1 strains showed stronger activity than the other strains under different chromium concentrations. When the Cr^6+^ concentration was 477.25 mg·L^−1^, the A1 adsorption rate was 44.31%; when the Cr^6+^ concentration was 48.9 mg·L^−1^, the A1 adsorption rate was as high as 65.75%. The sequencing results of *A. niger* are shown in Figure 1a. The phylogenetic analysis results are presented in Figure 1b.

### 3.2. A. niger Colony Characteristics under Chromium Stress

The *A. niger* community growth rates under stress from different chromium concentrations are shown in Table 2. In the first 24 h of culturing, the effect of chromium stress on *A. niger* colony growth was not evident, whereas it was significantly inhibited after 48 h. When the culturing time was 96 h, the colony diameter in the CK group reached 70.13 mm; the difference between treatments was significant (*p* < 0.05). When Cr^6+^ concentration was 500 mg·L^−1^, the diameter of a single colony was 41.03 mm. Although high Cr^6+^ concentrations evidently inhibited *A. niger* growth, strain A1 still showed strong activity. 

**Figure 2:**
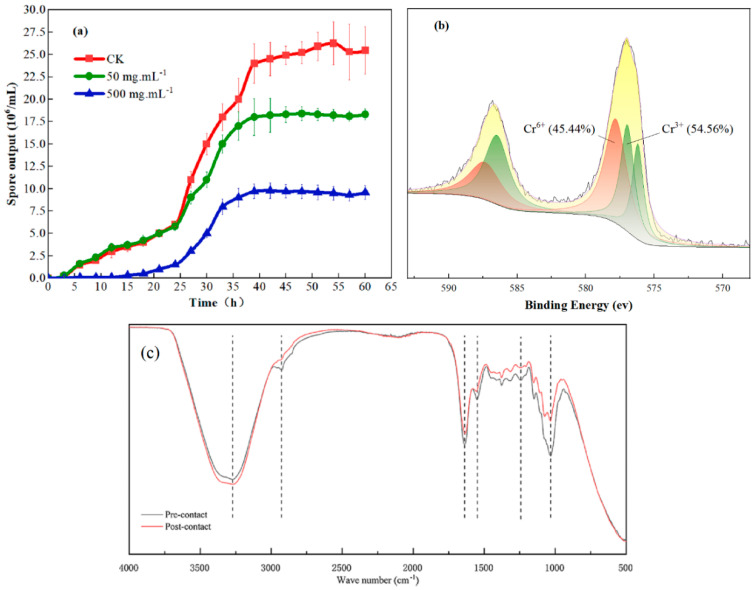
(**a**) Sporulation curves of *Aspergillus niger* strain A1 in chromium solution. CK—control. (**b**) X-ray photoelectron spectroscopy patterns of *A. niger* after 24 h of exposure to Cr^6+^. (**c**) Fourier transform infrared spectroscopy analysis of *A. niger* pre-contact or post-contact with Cr^6+^.

Figure 2a shows the effects of different Cr^6+^ concentrations on *A. niger* sporulation characteristics. When chromium concentration was low, sporulation ability did not show significant change in the first 24 h of culturing; however, in the 500 mg·L^−1^ Cr^6+^ treatment, sporulation ability was inhibited since the beginning of culturing. The experimental group exposed to 500 mg·L^−1^ Cr^6+^ entered the stable spore growth period at 36 h, and other treatments reached equilibrium after 39 h. During the whole process, A1 showed strong tolerance to both low and high Cr^6+^ concentrations. 

Figure 2b shows the peaks in the Cr 2p spectra of *A. niger* after contact with 50 mg·L^−1^ Cr^6+^. The peaks were located at 576.9 eV and 580.0 eV, indicating that Cr^6+^ and Cr^3+^ coexisted on the surface of *A. niger*. The results of peak separation showed that the two strong peaks were formed through the combination of five peaks. The peaks at binding energies of 587.5 eV and 577.9 eV corresponded to the existence of Cr^6+^, and the peak area accounted for 45.44%. The peaks at binding energies of 586.5 eV, 577.0 eV, and 576.2 eV corresponded to the existence of Cr^3+^, and the peak area accounted for 54.56%. XPS analysis directly proved that the *A. niger* membrane system had a fixation effect on Cr^6+^, and further indicated that *A. niger* has the ability to reduce Cr^6+^ to Cr^3+^. After 2d contact, more than half of the Cr^6+^ fixed by the cell surface was reduced to Cr^3+^.

The changes in functional groups on the surface of *A. niger* between post-contact and pre-contact with 50 mg·L^−1^ Cr^6+^ ions were determined via FTIR at wavelengths of 400–4000 cm^−1^ (Figure 2c). The peak difference in the two curves indicates the change in functional groups on the surface of *A. niger*. The peak near 3400 cm^−1^ was the stretching vibration of –OH. The peak at 3270 cm^−1^ shows the presence of the hydroxyl group and the stretching vibration of—NH_2_. The peak between 2930, 1680–1040 cm^−1^ shows the stretching vibration of -CH_2_, C=O, and C-O, respectively. The peak intensity of post-contact was higher than that of pre-contact, indicating that hydroxyl groups were barely involved in the chromium adsorption process. After contact, the peak at 2930 cm^−1^ disappeared, implying that the reaction consumed the methylene group (>CH_2_) from the surface of *A. niger*. The loss of peaks at 1640 cm^−1^ and 1550 cm^−1^ suggest that the glycoproteins on *A. niger*’s surface may have been involved in the chromium adsorption process. The peaks at 1000–1500 cm^−1^ were characteristic of carboxyl and amine groups. Consistent with the result of the XPS, FTIR analysis also revealed that the *A. niger* cell surface had great potential for Cr^6+^ adsorption. A variety of reductive groups on the membrane system are involved in the fixation and reduction of Cr^6+^.

### 3.3. Optimization of Adsorption Conditions

**Figure 3:**
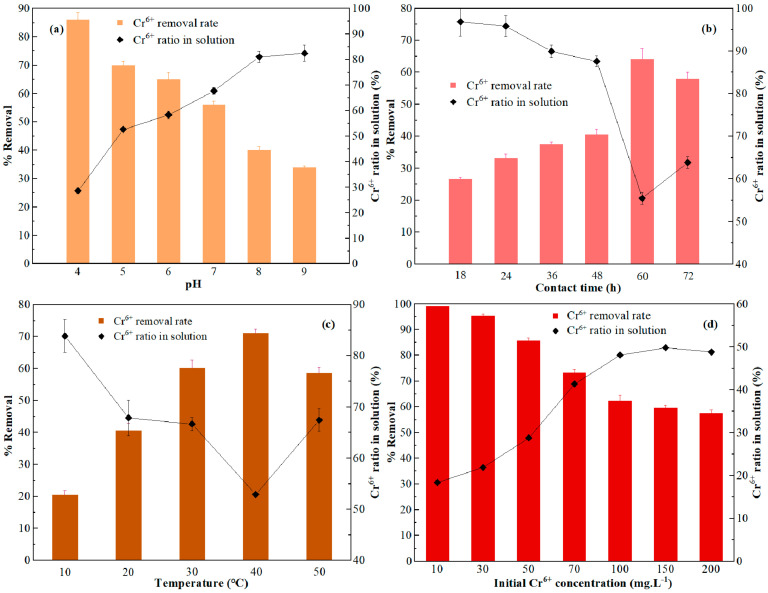
Influences of (**a**) pH, (**b**) contact time, (**c**) temperature, and (**d**) initial Cr^6+^ concentration on Cr^6+^ removal rate and Cr^6+^ ratio in the remaining solution.

When the culture temperature was 30 °C and the initial Cr^6+^ concentration was 50 mg·L^−1^, the Cr^6+^ removal rate decreased continuously and the Cr^6+^ ratio in solution increased continuously as pH increased from 4 to 9 (Figure 3a). The adsorption rate of Cr^6+^ and the reduction rate of Cr^3+^ at pH 4 were the highest (reaching 86% and 35%), and the percentage of Cr fixed by membrane and intracellular adsorption in *A. niger* reached 51%. According to the experimental results, *A. niger*(A1) had a better removal effect on Cr^6+^ in an acidic environment; at the same time, A1 can effectively promote the transformation of Cr^6+^ to low-toxicity Cr^3+^.

When the culture temperature was 30 °C, pH was 7, and the initial Cr^6+^ concentration was 50 mg·L^−1^, the adsorption effect of strain A1 on Cr^6 +^ increased with the increase in culture time within 0–60 h. (Figure 3b). The adsorption effect was the largest when the contact time was 60 h, at which time, the Cr^6+^ removal rate peaked at 64% and the Cr^6+^ ratio in solution reduced to 36%, and subsequently, the Cr^6+^ removal rate began to decline. At 60 h, *A. niger* adsorbed and fixed about 35% Cr, and the intracellular adsorption basically reached dynamic equilibrium. 

When pH was 7 and the initial Cr^6+^ concentration was 50 mg·L^−1^, the Cr^6+^ removal rate increased significantly when the temperature was increased from 10 °C to 40 °C (Figure 3c). When the temperature was 40 °C, the Cr^6+^ removal rate and adsorption rate of *A. niger* reached maximum values of 71% and 45.2%, respectively, and the Cr^6+^ ratio in solution reduced to 29%; subsequently, the Cr^6+^ removal rate began to decline as the temperature continued to increase. The optimum temperature range for Cr^6+^ adsorption by strain A1 was 30–40 °C. An increase in temperature within the suitable range can increase the Cr^6+^ removal capacity and the adsorption capacity of cells, but temperatures that are too high or too low will damage the cell structure or inhibit the activity of cells, resulting in a decrease in adsorption capacity.

When the culture temperature was 30 °C, pH was 4, and the initial Cr^6+^ concentration was 10 mg·L^−1^, the Cr^6+^ removal rate and adsorption rate of *A. niger* reached 99.1% and 95.1%, respectively, i.e., the lower the concentration, the higher the adsorption rate (Figure 3d). When Cr^6+^ concentration was 200 mg·L^−1^, the Cr^6+^ removal rate and adsorption rate of *A. niger* were 57.5% and 12.9%, respectively. Without a carbon source, the higher the initial Cr^6+^ concentration was, the more Cr^6+^ was converted into Cr^3+^ by *A. niger*. At the same time, the adsorption rate of *A. niger* to Cr decreased with the increase in the initial Cr^6+^ concentration in the solution, but there was no significant difference in the total adsorption amount. Therefore, with a thicker solution, the biomass of *A. niger* should be increased to ensure the adsorption effect.

### 3.4. Adsorption Kinetics and Isotherm Analyses of A. niger in Chromium Solution

**Figure 4:**
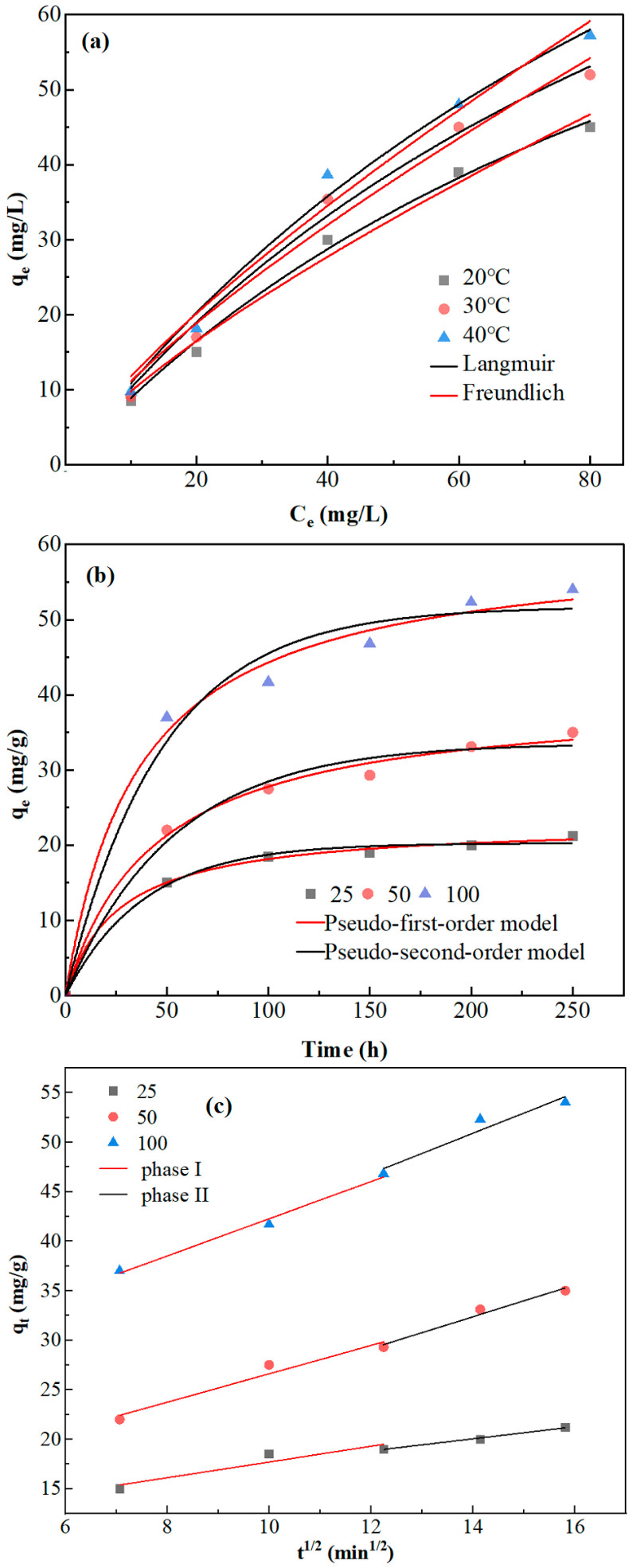
(**a**) Adsorption isotherm study for *Aspergillus niger*. (**b**) Kinetic analysis for *Aspergillus niger*. (**c**) The intra-particle diffusion model of *Aspergillus niger*.

To further determine the chromium adsorption capability, Langmuir (Equation (5)) and Freundlich (Equation (6)) isotherms were used to study the equilibrium data of adsorption at different temperatures (Figure 4a).

All values of the two isotherm constants are given in Table 3. The results indicate that the Langmuir isotherm model was most suitable for Cr^6+^ ion elimination onto *A. niger*. The Langmuir isotherm had a higher correlation coefficient (R^2^) and a better fitting effect. This indicated that the adsorption process of Cr^6+^ in water by *A. niger* was mainly monolayer adsorption. The Langmuir monolayer capacity (q_e_max) for Cr^6+^ at 40 °C was 117.71 mg·g^−1^ when the initial Cr^6+^ concentration was 50 mg·L^−1^.

The experimental data for the kinetic experiments were fitted with two adsorption kinetics models, i.e., pseudo-first-order and pseudo-second-order models, to explain the adsorption process between *A. niger* and Cr^6+^ (Figure 4b). The calculated kinetic parameters and R^2^ values are given in Table 4. Under different initial Cr^6+^ concentrations, the pseudo-second-order model had a higher correlation coefficient (R^2^) and a better fitting effect. The calculated values (q_e_cal) of the pseudo-second-order model were 38.353 mg/g and 57.282 mg/g when the initial Cr^6+^ concentrations were 50 mg·L^−1^ and 100 mg·L^−1^, respectively. These results revealed that adsorption kinetics could be described by pseudo-second-order kinetic models, suggesting that the process controlling the adsorption rate was complicated and may have been physico-chemical composite adsorption dominated by chemisorption.

In the intra-particle diffusion model, the straight lines did not pass through the origin (Figure 4c), indicating that intra-particle diffusion was not the only mechanism controlling the adsorption rate. The intra-particle diffusion model could fit two adsorption stages with different initial chromium concentrations. Two different adsorption rate constants for stepwise adsorption were obtained (Table 5). The results suggested that the diffusion rate of Cr^6+^ into *A. niger* was very slow and was restricted by other factors.

### 3.5. Remediation of Chromium Pollution in Red Soil by A. niger (Strain A1)

**Figure 5:**
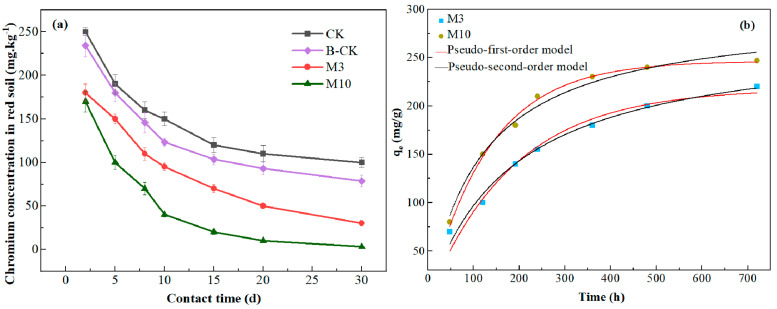
(**a**) Cr^6+^ concentration in red soil with increased contact time. CK—control; B-CK—commercial microbial agent. (**b**) Kinetic analysis for Cr^6+^–*Aspergillus niger*.

The change in soil chromium content in each treatment was continuously measured for 30 d (Figure 5a). Compared with CK, *A. niger* inoculation significantly promoted reductions in chromium content in red soil. Compared with B-CK, improving the biomass of indigenous *A. niger* achieved a better removal effect of Cr^6+^ than inoculation with the same amount of exogenous microbial agent. After only 2 d of treatment, the Cr^6+^ content in M3 and M10 soils had decreased to 180 mg·kg^−1^ and 175 mg·kg^−1^, respectively. The results showed that the amount of *A. niger* positively correlated with the remediation effect of chromium pollution in red soil. After 30 d of microbial remediation, the chromium concentration in red soil decreased from 300 mg·kg^−1^ to < 50 mg·kg^−1^, demonstrating a strong remediation effect.

The chromium adsorption curves of *A. niger* were calculated using two kinetic models (Figure 5b), and the calculated kinetic parameters and R^2^ values are given in Table 6. The pseudo-second-order model had a higher correlation coefficient (R^2^) and a better fitting effect in M3. However, the pseudo-first-order model had a higher correlation coefficient (R^2^) and a better fitting effect in M10, and the theoretically calculated value (q_e_cal) of the pseudo-first-order model was closer than the experimental results (q_e_exp), but lower. The q_e_cal calculated by the pseudo-second-order model was much higher than the q_e_exp. The results showed that the removal process of Cr^6+^ may be a physico-chemical composite process dominated by physical adsorption.

The intra-particle diffusion model was used to fit the data of the two experimental groups, and two adsorption stages were obtained. The adsorption rate constants for stepwise adsorption were obtained (Table 7). The straight lines did not pass through the origin, indicating that the intra-particle diffusion model was not the only mechanism controlling the adsorption rate. In the first stage, the internal diffusion rates of M3 and M10 were similar, whereas the internal diffusion rate of M3 was higher in the second stage. 

**Figure 6:**
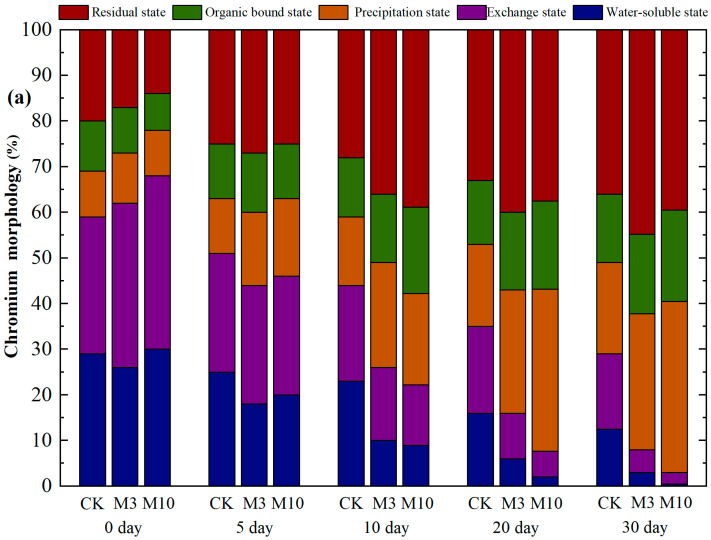

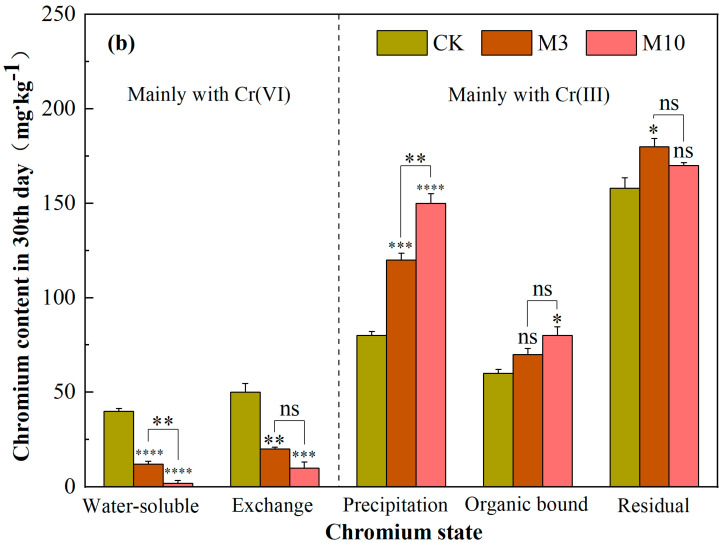
(**a**) *Aspergillus niger* drives the transformation of chromium form in red soil. (**b**) Chromium distribution in soil after *A. niger* inoculation. CK—control. (****, *p* < 0.0001; ***, 0.0001 < *p* < 0.001; **, 0.001 < *p* < 0.01; *, 0.01 < *p* < 0.05; ns, no significant difference).

After the screened strain A1 was inoculated in the red soil with severe chromium pollution (300 mg·kg^−1^ chromium), samples were taken every 5 d, and the morphological distributions of chromium in the red soil under various treatments were continuously measured for 30 d using a sequential extraction method (Figure 6a). The forms of chromium in soil were divided into: water-soluble, exchange, precipitation (Fe-Mn combined), organic-bound, and residue states. Among them, the water-soluble and exchange states have strong migration ability, are highly toxic, and are easily absorbed by organisms, mainly Cr^6+^; meanwhile, the precipitation, organic-bound, and residue states are more stable, their direct use by organisms is more difficult, and most of them are Cr^3+^. At the beginning of the experiment, water-soluble and exchange states were the main components of the three treatment groups. In M3 treatment, highly toxic forms accounted for >60%, while in M10 treatment, they accounted for >65%. The transformation rate of chromium from high-toxicity to low-toxicity forms in M3 and M10 was significantly increased after inoculation with *A. niger;* the transformation rate positively correlated with the inoculation amount. After only 5 d of treatment, the proportion of the highly toxic form in M3 and M10 was reduced to <50%, which was lower than that in CK. After 30 d of treatment, the proportion of the highly toxic form in CK was still nearly 30%, whereas it was <10% in M3 treatment and <5% in M10 treatment. The contents of various forms of chromium in red soil on the 30th day are shown in Figure 6b. After 30 d of treatment, the concentration of chromium in a residual state in CK increased to 170 mg·kg^−1^. The concentration of chromium in a residual state increased by 16%, and the concentration in the precipitation state increased by 10% after 30 d of treatment. However, in M3 and M10 inoculated with *A. niger*, the concentrations of the residual and precipitation states increased significantly; the concentrations of the residual state increased to 180 mg·kg^−1^ and 158 mg·kg^−1^ after 30 d, while those of the precipitation state increased to 80 mg·kg^−1^ and 150 mg·kg^−1^, and those of the organic-bound state increased to 60 mg·kg^−1^ and 80 mg·kg^−1^, respectively. During the experimental period, by increasing indigenous *A. niger* biomass, the Cr in red soil was transformed from a highly toxic state to a less toxic state; toxic Cr^6+^ was largely removed and Cr migration capacity greatly decreased. 

**Figure 7:**
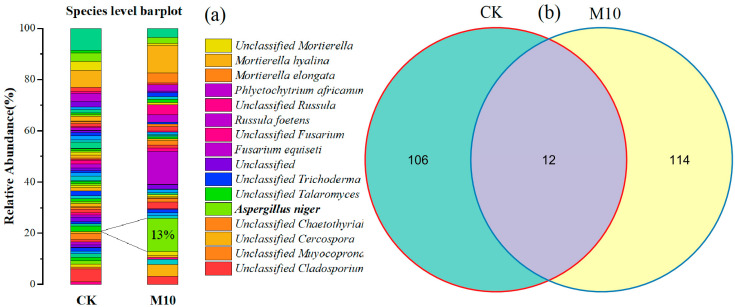
(**a**) Variation of soil fungi relative abundance at species level after 30 d of *A. niger* inoculation. (**b**) OTUs—Venn diagram analysis.

High-throughput sequencing was used to analyze soil microbial diversity in M10 and CK treatments after 30 d of *A. niger* inoculation. The results showed that the inoculation of *A. niger* significantly increased the abundance of *A. niger* in red soil with severe chromium pollution (Figure 7a). At the same time, after *A. niger* was inoculated, the total amount of fungi in the red soil did not change much, but the species changed greatly (Figure 7b).

## 4. Discussion

### 4.1. Significance of Screening Indigenous Chromium-Tolerant Microorganisms to Treat Heavy Metal Pollution in Red Soil

In recent years, red soil has been seriously degraded, increasing ecological problems [30], and the application of conventional physical and chemical methods has had limited success. In contrast, microbial remediation is environmentally friendly and pollution-free, and highly suitable for remediating heavy metal pollution in red soil. Owing to the bioclimate in red soil, colonization by exogenous microorganisms is difficult after inoculation. This is a considerable problem while using microorganisms to remediate heavy metal pollution in red soil, which directly affects the remediation [31]. Thus, we isolated and screened indigenous chromium-resistant *A. niger* from chromium-polluted red soil using heavy metal chromium as an ecological filtration factor. After sufficient theoretical verification, *A. niger* screened in red soil was used for the treatment of chromium pollution in it, and encouraging results were obtained. The inoculation of indigenous *A. niger* could not only protect the microbial diversity of red soil, but also significantly increase the abundance of *A. niger* and enhance the ability of Cr passivation. These results proved the feasibility of activating indigenous microorganisms to deactivate soil heavy metals; at the same time, the problem of the unstable effect of exogenous microorganisms was also solved in this way.

In this study, the colony characteristics of strain A1 screened from red soil under Cr stress were studied, which proved that strain A1 could tolerate high concentrations of Cr^6+^ and had the capacity to adsorb Cr^6+^. On this basis, the effects of temperature, pH, initial Cr^6+^concentration and contact time on Cr^6+^adsorption by strain A1 were discussed in terms of condition optimization. It is proven that Indigenous *A. niger* exhibited the highest adsorption capacity under acidic conditions and was highly adaptable to medium and high ambient temperatures. The selected indigenous *A. niger* demonstrated strong potential for the remediation of chromium pollution in red soil in warm climates. 

### 4.2. Potential of Indigenous A. niger to Reduce Chromium Pollution in Red Soil Environments and Its Adsorption Mechanism

The main functions of microorganisms for alleviating heavy metal pollution can be categorized as: reducing intake, increasing excretion, and metabolic detoxification. The use of gene editing to knock out specific channel protein genes can produce microorganisms that are hyperaccumulators of specific heavy metals [32]. Strengthening the metabolism and detoxification ability of microorganisms can effectively improve their heavy metal adsorption abilities, and the stress caused by heavy metals can induce them to produce many functional proteins [33]. These proteins have strong binding ability for cadmium, copper, mercury, chromium, and other metals, and can transform water-soluble heavy metals into less toxic precipitated and organic-bound states, thereby reducing metal bioavailability in cells [34]. The *A. niger* isolated and purified in the present study has previously been used to remove heavy metals from sewage [35]. Recent studies show that chitosan or other extracellular polymeric substances (EPS) on the cell wall of *A. niger* can cooperate with various functional groups to complete the extracellular adsorption of heavy metal ions [36,37,38]. In this study, FTIR analysis showed that many reducing groups on the surface of *A. niger* were involved in the fixation of Cr^6+^, such as -C=C-, -C=0, -C-O-C, and -NH_3_. In order to explore the binding form of chromium on *A. niger*’s surface, we compared the differences between extraction with 8000 rpm centrifugation and filtration, and the results showed that the chromium content of centrifugal extraction was slightly lower than that of filtration extraction. Combined with XPS analysis, this shows that *A. niger*’s membrane system can fix Cr^6+^ with a relatively firm structure (surface complexation) and reduce it to Cr^3+^ in large quantities, except that a small amount of Chromium is attracted by ion exchange. In the subsequent supplementary experiment, the filtered *A. niger* bodies were broken to compare the amount of chromium fixed by the membrane system and absorbed by the cytoplasm. The results showed that the main way for *A. niger* to absorb chromium is through intracellular adsorption, and the surface adsorption amount is positively correlated with the biomass of *A. niger*; moreover, there was little difference in the fixation capacity per unit membrane area. There is also an upper limit for intracellular adsorption. When the intracellular chromium content is too high, *A. niger* can reduce the intake of chromium by closing the transport channel, which is also reflected in the kinetic analysis of this study. In this study, three kinetic models (including the intra-particle diffusion model) were used to elucidate the adsorption process of *A. niger* in solution and soil. The fitting results of the intra-particle diffusion model show that chromium could not diffuse through *A. niger* membranes and required endocytosis or active transport to enter the cell; in the early stage of adsorption, it was mainly the surface adsorption of membrane system, and the internal diffusion rate was very slow. In the middle stage of adsorption, the surface adsorption reached a dynamic balance and the internal diffusion effect was enhanced; additionally, detoxification by *A. niger* occurred through intracellular metabolism, and it stored chromium in the cells. At the late stage of adsorption, there was no internal diffusion effect, the absorption rate of chromium by *A. niger* was equal to the discharge rate or *A. niger* stopped absorbing chromium, and the adsorption effect reached a dynamic balance. Based on the other analysis, we found that the increase in Cr^6+^ concentration could improve the chromium adsorption rate of *A. niger*; however, the increase in the adsorption rate was limited by the combined action of the strong driving force of high concentration and binding sites on the surface of *A. niger*. These conclusions improve on the theory proposed by Chhikara [39].

In the red soil environment, a low dose of *A. niger* inoculation was more consistent with the intra-particle diffusion model. This is most likely because the complex soil environment can passivate chromium in addition to *A. niger*; therefore, when *A. niger* concentration was low, the adsorption process was not dominated by *A. niger* only. Compared with the microbiological agents purchased on the market, the application of the same amount of *A. niger* achieved significantly better results. Commercial microbial agents had a good effect within 7–10 d after inoculating, and then, the effect weakened. This phenomenon may be related to the difficulty in the colonization of exogenous microorganisms or the inadaptability of exogenous microorganisms to the acidic red soil environment. However, by increasing the biomass of *A. niger*, the passivation effect of hexavalent chromium in red soil was significantly improved, and the passivation rate was positively correlated with the addition amount.

As *A. niger* has a certain enrichment effect of chromium from the environment, in follow-up research, it could be transformed into a chromium hyperaccumulator through gene elimination and destruction of the channel protein synthesis gene that pumps out chromium. In addition, the results of the optimization study indicated that the optimal pH for chromium pollution remediation using *A. niger* was consistent with the main pH distribution of red soil, and that *A. niger* could drive the transformation of water-soluble and exchangeable states of chromium to precipitation and residue states while absorbing soil chromium. Moreover, its addition directly affected the rate of transformation of chromium state and greatly reduced the toxicity of chromium in red soil. In this study, the potential for indigenous *A. niger* to be used in treating heavy metal pollution in red soil was verified in many aspects. At the same time, this study proved that the activation of indigenous microorganisms was more effective than adding exogenous microorganisms, and the mechanism of chromium adsorption by *A. niger* was studied. Based on these conclusions, we can further put forward various improvement measures to improve the effectiveness of applications of indigenous *A. niger*.

### 4.3. Contributions and Limitations of Current Research and Future Development Directions

The method used in this study can not only be used for the remediation of chromium pollution in red soil but also for the in situ remediation of other soil types and heavy metal elements by screening native strains with high tolerance. Furthermore, similar research can be applied to the current treatment of organic pollutants and soil microplastics. After screening the functional strains for pollutants, pollutant degradation can be achieved by activating or externally supplementing indigenous microorganisms. Organic pollutants can form non-toxic metabolites after being degraded by microorganisms and return to the carbon and nitrogen cycles [40]. Therefore, the activation of indigenous microorganisms is more suitable for the treatment of organic pollutants than for heavy metal pollution, considering the recovery of pollutants from the red soil.

This study showed that the use of indigenous *A. niger* to remediate soil chromium pollution has high feasibility and low cost, is convenient, has a stable effect, and produces no secondary pollution. However, microbial remediation, as with traditional remediation methods, faces the problem that the treated chromium remains in the soil. There is a risk that chromium may be re-released under certain circumstances after being adsorbed into cells or transformed into a less toxic form. On this basis, through the immobilized *A. niger* method, further research on technology for the recovery of bioremediation can effectively solve these problems. In addition, the soil’s microenvironmental stability is a dynamic equilibrium. In soil, plants, microorganisms, and other organisms can maintain the relative stability of their internal chemical composition by adjusting the concentration of chemical elements and the measurement ratio of different chemical elements in the body to cope with the influence of external conditions such as soil nutrient limitation on their own growth. This mechanism is called homeostasis [41]. After the addition of exogenous strains, the biomass would be greatly increased in a short time; however, the soil bacterial colony structure would eventually be stabilized. The present study found that the excessive addition of *A. niger* significantly improved the treatment effect in a short period. However, the influence of *A. niger* on adsorption gradually decreased with the stabilization of soil bacterial colony structure. Therefore, there may be a potential upper limit of bioremediation, which may be related to the colony characteristics and niche competitiveness of the selected microorganisms. Further research is necessary to solve this problem, and to improve bioremediation via strain improvement or gene editing.

### 4.4. Virulence Test

**Figure 8:**
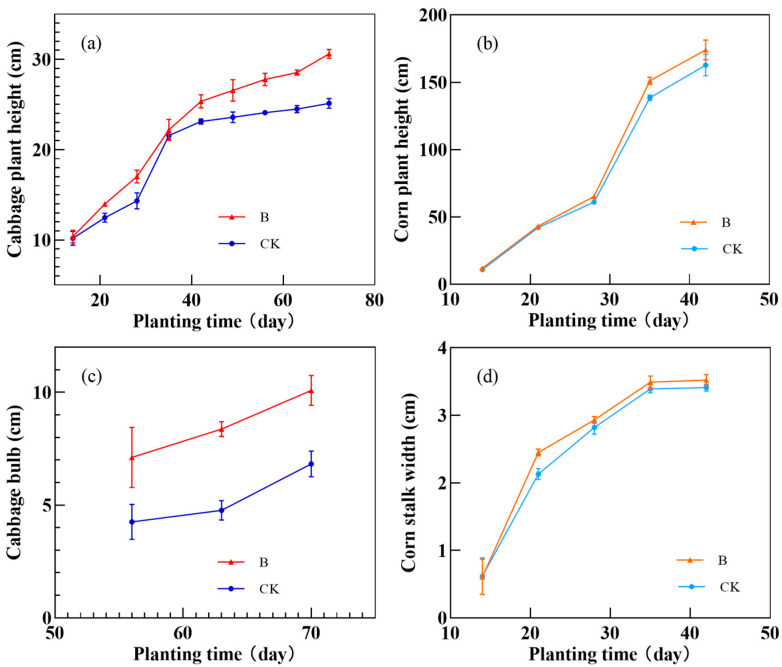
(**a**) Height of cabbage plant; (**b**) height of corn plant; (**c**) bulb of cabbage plant; (**d**) width of corn plant.

*Aspergillus niger,* which was screened, is one of the most common molds in daily life. It is widely distributed in soil and air, and is also widely used in the treatment of heavy metal pollution, making it valuable for use in the actual environment. However, virulence tests still need to be carried out before using *A. niger* as a fungal agent to control heavy metal pollution in red soil. For this reason, we inoculated *A. niger* into corn and cabbage fields, and continued to observe the effects of *A. niger* inoculation on the growth of corn and cabbage for 2 months. The observation results are shown in Figure 8.

Cabbage and corn are the representative crops of Cruciferae and Gramineae, respectively. The observation results showed that the inoculation of *A. niger* had no significant adverse effect on crop growth. Therefore, the use of indigenous *A. niger* to control chromium pollution in red soil is a safe and green new scheme.

## 5. Conclusions

An indigenous chromium-resistant *A. niger* strain was screened from red soil. The strain showed high activity with exposure to 500 mg·L^−1^ of Cr^6+^. *Aspergillus niger* had a strong and rapid adsorption effect on Cr^6+^ ions under acidic conditions at 20–50 °C. Our investigations pertaining to its adsorption mechanism showed that *A. niger* mainly adsorbed chromium through intracellular adsorption, surface complexation, and ion exchange. Extracellular adsorption is accomplished through the cooperation of EPS and various functional groups on the cell surface. Complete adsorption on the surface was followed by intracellular metabolic detoxification, and it was evident that this effect would be continuously improved with an increase in biomass after colonization by *A. niger*. A large amount of chromium was enriched into *A. niger* cells because it was restricted from freely crossing the surface of *A. niger* cells, consequently reducing the available chromium content in soil. Moreover, the inoculation of indigenous *A. niger* can greatly improve the dominance of *A. niger* in red soil, drive the transformation of a large amount of chromium into a less toxic form, and greatly reduce the content of Cr^6+^. In conclusion, the proposed method of application of indigenous *A. niger* to treat chromium pollution in red soil is a feasible strategy, and the effect is better than using traditional exogenous microbial agents.

## Figures and Tables

**Table 1:** Screening of chromium tolerance of *A. niger* strains.

Strain	Initial Cr^6+^ Concentration: 50 mg·L^−1^			Initial Cr^6+^ Concentration: 500 mg·L^−1^		
	Cr^6+^ Concentration after Culture (mg/L)	Adsorption Capacity (mg/L)	Adsorption Rate (%)	Cr^6+^ Concentration after Culture (mg/L)	Adsorption Capacity (mg/L)	Adsorption Rate (%)
CK	48.9	——	——	477.25	——	——
A1	10.93	37.97 ± 0.73 h	77.64	265.8	211.46 ± 0.94 f	44.31
A2	25.51	23.39 ± 0.44 g	47.83	353.05	124.29 ± 0.26 e	26.02
A3	34.05	14.85 ± 0 f	30.36	446.75	30.28 ± 0.18 d	6.39
A4	37.42	11.48 ± 0.11 d	23.47	453.5	23.81 ± 0.12 c	4.98
A5	43.17	5.73 ± 0.29 b	11.71	477.22	——	——
A6	36.31	12.59 ± 0.12 e	25.74	457	20.82 ± 0.26 b	4.24
A7	44.99	3.91 ± 0.09 a	7.99	477.3	——	——
B1	36.57	12.33 ± 0.08 e	25.21	460	17.35 ± 0.88 a	3.61
B1	34.15	14.75 ± 0.11 f	30.16	447	30.29 ± 0.11 d	6.33
B3	40.65	8.25 ± 0.13 c	16.87	458.85	18.50 ± 0.35 a	3.86

Note: Data are mean values ± SE. Different lowercase letters in the same column indicate significant differences determined by Duncan’s multiple range test (*p* < 0.05). CK—control.

**Table 2:** Single colony diameters of *A. niger* strain A1 during exposure to different Cr^6+^ concentrations.

Initial Chromium Concentration (mg/L)	Single Colony Diameter (mm)			
	24 h	48 h	72 h	96 h
CK	4.50 ± 0.29 b	17.16 ± 0.17 d	30.08 ± 0.08 e	70.13 ± 0.08 e
50	3.83 ± 0.16 ab	16.16 ± 0.16 c	27.25 ± 0.14 d	65.18 ± 0.09 d
100	3.91 ± 0.08 ab	15.25 ± 0.14 b	24.13 ± 0.08 c	58.56 ± 0.29 c
200	4.50 ± 0.22 a	15.00 ± 0.14 b	22.21 ± 0.06 b	50.80 ± 0.60 b
500	3.66 ± 0.22 a	14.00 ± 0.00 a	19.51 ± 0.13 a	41.03 ± 1.18 a

Note: Data are mean values ± SE. Different lowercase letters in the same column indicate significant differences determined by Duncan’s multiple range test (*p* < 0.05). CK—control.

**Table 3:** Fitting constants of two isotherm models for different adsorbents.

Temperature (°C)	Langmuir Model			Freundlich Model		
	q_e_max(mg/L)	K_L_(L/mg)	R^2^	K_F_((mg/g)/(L/mg)^(1/n)^)	n	R^2^
20	95.13	0.0085	0.9929	1.7261	1.3288	0.9810
30	104.80	0.0083	0.9882	1.9242	1.3129	0.9736
40	117.71	0.0076	0.9879	1.9676	1.2878	0.9757

**Table 4:** Fitting parameter values of the two kinetic models.

Initial Cr^6+^	q_e_exp (mg/g)	Pseudo-First-Order Model			Pseudo-Second-Order Model		
Concentration (mg/L)		q_e_cal (mg/g)	K_1_ (h^−1^)	R^2^	q_e_cal (mg/g)	K_2_ (g/mg/h)	R^2^
25	21.2	19.83	0.0258	0.9927	22.941	0.00165	0.9974
50	35.1	32.25	0.0188	0.9825	38.353	0.00057	0.9934
100	54.0	49.45	0.0212	0.9745	57.282	0.00046	0.9892

**Table 5:** Fitting constant values of intra-particle diffusion model.

Initial Cr^6+^	Intra-Particle Diffusion Model			
Concentration (mg/L)	K_i1_ (g/mg/min1/2)	R^2^	K_i2_ (g/mg/min1/2)	R^2^
25	0.79366	0.78666	0.61527	0.98419
50	1.43346	0.9141	1.60844	0.95323
100	1.87709	0.98207	2.04	0.86863

**Table 6:** Fitting parameter values of the two kinetic models.

Process Mode	q_e_exp (mg/g)	Pseudo-First-Order Model			Pseudo-Second-Order Model		
		q_e_cal (mg/g)	K_1_ (h^−1^)	R^2^	q_e_cal (mg/g)	K^2^ (g/mg/h)	R^2^
M3	220	208.14	0.00539	0.96344	259.66	0.0002	0.98527
M10	247	242.03	0.0076	0.99297	284.04	0.00028	0.98461

**Table 7:** Fitting constant values of the intra-particle diffusion model.

Process Mode	Intra-Particle Diffusion Model			
	K_i1_ (g/mg/min1/2)	R^2^	K_i2_ (g/mg/min1/2)	R^2^
M3	12.30598	0.9819	5.7385	0.96802
M10	12. 87473	0.94838	3.17912	0.84897

## Data Availability

Not applicable.

## Abbreviations

ddH_2_O—double distilled water; FTIR—Fourier transform infra-red spectroscopy; PCR—polymerase chain reaction; XPS—X-ray photoelectron spectroscopy; EPS— extracellular polymeric substances; SDYA—a medium used to isolate and culture fungi, Sabouraud Dextrose Agar with Yeast Extract.
